# What is the care economy? A scoping review on current evidence, challenges, facilitators and future opportunities

**DOI:** 10.3389/fpubh.2025.1540009

**Published:** 2025-05-14

**Authors:** I. Blackberry, J. Boak, K. Barclay, H. Khalil

**Affiliations:** ^1^Care Economy Research Institute, La Trobe University, Albury-Wodonga, VIC, Australia; ^2^John Richards Centre for Rural Ageing Research, La Trobe Rural Health School, La Trobe University, Albury-Wodonga, VIC, Australia; ^3^School of Psychology and Public Health, Department of Public Health, La Trobe University, Melbourne, VIC, Australia

**Keywords:** care economy, workforce, caregiving, gender equity, informal care, care, health, social

## Abstract

**Background:**

The care economy gained its prominence during the COVID-19 pandemic. The value and impact of caregiving, mostly shouldered by women, was not as visible until such crisis point. Health care and social support sectors represent the largest and fastest growing industry globally. This scoping review aims to elucidate the current state of play in the care economy, where there is a great reliance on informal and formal care workforce to deliver care for populations across all age groups and abilities.

**Methods:**

Following Joanna Briggs Institute (JBI) methodology and PRISMA-SCR reporting guidance, we searched MEDLINE, Embase, CINAHL, PsycINFO, Campbell collaboration database, Social Science Abstracts, Library and Information Science Abstracts (LISA) and Scopus. Quantitative and qualitative original research on disability, aged care, early childhood education and care, rural, veterans, migrants and informal and formal care workforce from January 2018 until November 2023 were examined.

**Results:**

Of 354 studies selected, 20% were from the United States of America, 11% each were from China and the United Kingdom. Most studies employed cross-sectional design. A quarter of the studies included adults aged 65 years and above while 6% were adults aged 18 to 64 years. These age groups combined were included in an additional 27% of studies. Women were overrepresented in 70% of the studies. Nearly two-thirds of caregivers were spouses or partners. Barriers to providing care were lack of education, support and monitoring of caregiver well-being, loss of income or ability to earn money, reduced social life and increased out-of-pocket costs. Gaps in research included migrant populations’ contribution to the care economy, gender and diversity inequality in the care economy. The care economy could be improved through providing education for caregivers, care workforce engaging with caregivers in the care plan, and governments’ overhaul of compensation for caregivers through direct financial support and employment benefits.

**Conclusion:**

The care economy is an emerging research area. There continues to be a paucity of research evidence across some geographical areas. Studies are mostly short term or small scale with very little evidence around the value of care. Given the growing aging population, more research is needed to elucidate the positive aspects of caring by formal and informal care workforce to the population, society and economy.

**Protocol registration:**

The protocol is registered with Open Science Framework (10.17605). “Definitions, key themes and aspects of the care economy-a scoping review protocol,” https://osf.io/ypmuh.

## Introduction

The world is experiencing a major demographic shift. In 2020, the number of older adults outgrew children under 5 years old for the first time (1). As life expectancy increases and the fertility rate declines, there is a growing aging population requiring both formal and informal care. Studies have shown that for some people seeking care and their caregivers, there is a degree of reluctance to access support services and reach out to their care support network. There is a stigma associated with seeking care, being seen as a failure among others (2). King et al. (3) stated that “Care work has been accorded relatively low political priority because of the prevailing belief that caregiving is primarily the responsibility of the family and has little impact on economic development and growth” (p. 12).

The care economy is one of the fastest growing economic sectors globally, shaped by the capacities and dynamics between private, public, and community sectors (4). For example, formal care is estimated to be worth $648 billion in the United States (5) while in Australia, the care economy is the largest and fastest growing industry in health care and the social support workforce (6). A report from the International Labor Organization (ILO) showed that informal care represented by nearly 2 billion people, accounts for 9% of global GDP, which is equivalent to USD $11 trillion (1). In Latin America, unpaid care contributes up to 24.2% of regional GDP (7).

The care economy encompasses all essential social, health, and well-being support services provided to individuals of all ages, abilities, and diverse backgrounds. It emphasizes the lived experiences of those involved in care, highlighting both the challenges and benefits of caregiving for individuals, communities, and broader society. Caregiving is crucial for enhancing workforce productivity and future-proofing economic growth, while also serving as a foundation for advancing systemic gender equality (8).

The care economy offers significant potential for job creation and economic growth. Investing in care as an economic priority is crucial for addressing large-scale economic challenges and promoting growth and equity. Accurate data can guide investments in areas like early childhood education and care (ECEC), care for older people, and healthcare, generating millions of jobs and supporting economic resilience. A World Economic Forum model projected that a USD $1.3 trillion investment in social jobs could yield $3.1 trillion in GDP and create 11 million jobs (9).

Policies that facilitate the care economy as a country priority are required to ensure the sustainability of caregiving as the cost of care becomes unsustainable, skilled workforce shortages increase and greater expectations of the quality of care received. The COVID-19 pandemic was instrumental in highlighting the importance of the care economy (10). In Australia in 2023, the government released a Care and Support Economy Roadmap (11) and undertook various Royal Commissions and Inquiries (12). Similarly, across Asia, the Malaysian, Brunei and Singapore governments released their care economy strategies (13).

The care sector was largely invisible to the role that women played in caring (14, 15). Care sector jobs are in high demand, driven particularly by the growing aging population (16). As such, the care industry is a significant contributor to employment, economic growth and societal well-being, and includes both formal and informal work (17). Focusing on the care economy can enhance social well-being, promote social mobility, and create more equitable opportunities for marginalized groups, particularly informal caregivers (17).

The care economy is important for gender equality (18, 19), socioeconomic equality, poverty reduction, inclusive growth and sustainable development (20). While the care economy has been discussed recently in policies from the United States (21) to Australia (22) and in the literature over the last decade, the concept remains broad and variably defined (23).

The aim of this study was to scope and map the current state of the global care economy initiatives, to identify conventions and patterns in the scholarly treatment of the concept of care economy across an individual’s life course. In addition to identifying aspects of care economy, we examined barriers and facilitators to care provision and identify recommendations for future research.

## Methods

This scoping review is based on the JBI methodology publications by Peters et al., 2020 and Khalil et al., 2016 (24, 25) The protocol is registered in Open Science Framework (registration number 10.17605) using PRISMA-SCR for reporting guidance (26). We acknowledge that the context of the care sectors across the life course is broad and may vary between countries. For this paper, we employed the Australian National Skills Commission’s Health Care and Social Assistance jobs classification being hospitals, other social assistance services, allied health services, medical services, residential care services, ECEC services, pathology and diagnostic imaging services, and other health care services (12). The protocol is registered in Open Science Framework (registration number 10.17605) using PRISMA-SCR for reporting guidance (26). We acknowledge that the context of the care sectors across an individual’s life course is broad and may vary between countries.

The database search included both peer reviewed quantitative and qualitative studies with the initial dates from January 2018 until 14th November 2023. This date range was aimed to provide contemporary research that covered pre-COVID-19 (pandemic start March 2020 and end May 2023) (27, 28). No gray literature was searched, as we were interested in original research that was published in peer-reviewed journals based on scientific methods that use evidence to develop conclusions. Systematic reviews, meta-analyses, meta-syntheses, scoping reviews and protocols were also excluded, as were studies not published in English.

### Data sources

A three-step search strategy was utilized in this review. An initial limited search of Ovid MEDLINE, followed by analysis of the text words contained in the title and abstract, and of the index terms used to describe the article. A second search using all identified keywords and index terms was undertaken across all included databases. The following databases were searched: Ovid MEDLINE, Embase, CINAHL, PsycINFO, Campbell collaboration database, Social Science Abstracts, Library and Information Science Abstracts (LISA) and Scopus (24). The search terms are shown in Table 1. Searches for all the databases are included in Appendix 1.

**Table 1:** Search terms.

Concept 1	Concept 2	Sector	Population	Care type
Care	Economy	Healthcare.mp.	CALD	Physical.mp.
Support	Value	Social.mp	ATSI	Psychological.mp.
Service	Productivity	time.mp. unpaid work.mp. paid work.mp. Human capabilit$.mp. health.mp. aged.mp. childcare.mp. Early childhood education.mp. Rural.mp. veteran$.mp. or Veterans/ Workforce/ or Health Workforce/ or workforce.mp. labour.mp. disabilit$.mp. employment.mp. welfare.mp. Palliative.mp. End of life Formal Informal Volunteer Lifespan/life course Preventative Chronic disease Family service Family violence Foster care Community Health system Primary.mp drug$ Adj alcohol Addiction Social adj housing Juvenile adj justice Kinship Work from home Social infrastructure Non market work	disab* migrant family carer veteran incarcerated volunteer* (infant or young or adult or adolescent) ADJ3 child* prime or working Adj age aged OR older adults OR Recipient Grandchild* Grandmother or grandparent*	Emotional.mp. Mental health Social wellbeing/well-being/well being

### Study selection

Articles from the 1st of January 2018 to the 14th of November 2023 were included in this review. This provided the opportunity to capture contemporary evidence published before, during and post-pandemic and reflect current state concerning the care economy, characteristics, challenges, facilitators and gaps.

The articles were imported to Covidence software for screening and data extraction. Screening steps were undertaken by two reviewers (Title and abstract screening: KB, MG, SK. Full-text screening: KB, JB and RG). HK and IB reviewed articles that required a third reviewer for agreement. The screening groups met regularly to review and discuss any discrepancies and to reach agreement on study inclusion, and to cross-check a 10% allocation of articles for quality review.

### Data extraction

Relevant data were extracted from the included studies to address the review questions using the methodology outlined by Peters et al. (24) and was conducted by KB, JB, RG. We used the ILO definition of the care economy to guide us with the data extraction, stating “*the care economy entails a diversified range of productive work with both formal and informal work activities for providing direct and indirect care necessary for the physical, psychological, social wellbeing of primarily care dependent groups such as children, the older adults, disabled and ill, as well as for prime-age working adults*.” (29). The data extraction group met regularly to discuss and resolve any discrepancies.

The data extracted included the following: author(s), year of publication, origin/country, aim of the study, study design, age of person receiving care, age of caregiver, gender, caregiver relationship, care setting, nature of care giving, length of caregiving, type of care delivery (direct/indirect) formal and informal work, who was funding the care, how the care was quantified and its measures, any roles of migrants and other types of caregivers in providing care. Barriers and facilitators to care provision and any gaps to care provision were identified from the discussion and conclusion included in the studies. Key terms for the data extraction were defined and are provided in Appendix 2.

### Data synthesis

We used an inductive approach for data extraction identifying characteristics of caregivers and recipients, aspects of care economy (quality of life, support, and workforce), contexts (i.e., children, aged health), barriers and facilitators to care provision highlighting any gaps and future research (30). Data extraction was conducted using Covidence systematic review software, which was then exported to NVivo software, where qualitative data were categorized and the use of word frequency contributed to initial coding. The codes for barriers and facilitators to care provision were then grouped to form themes. The main file was checked for the context of each word. Using this method, we attempted to minimize the duplication of word frequencies. The extracted data were represented in a logical and descriptive summary that aligned with the objective of the review. Due to the large number of studies included, each study was labeled with a unique identification number [ID x].

## Results

**Figure 1:**
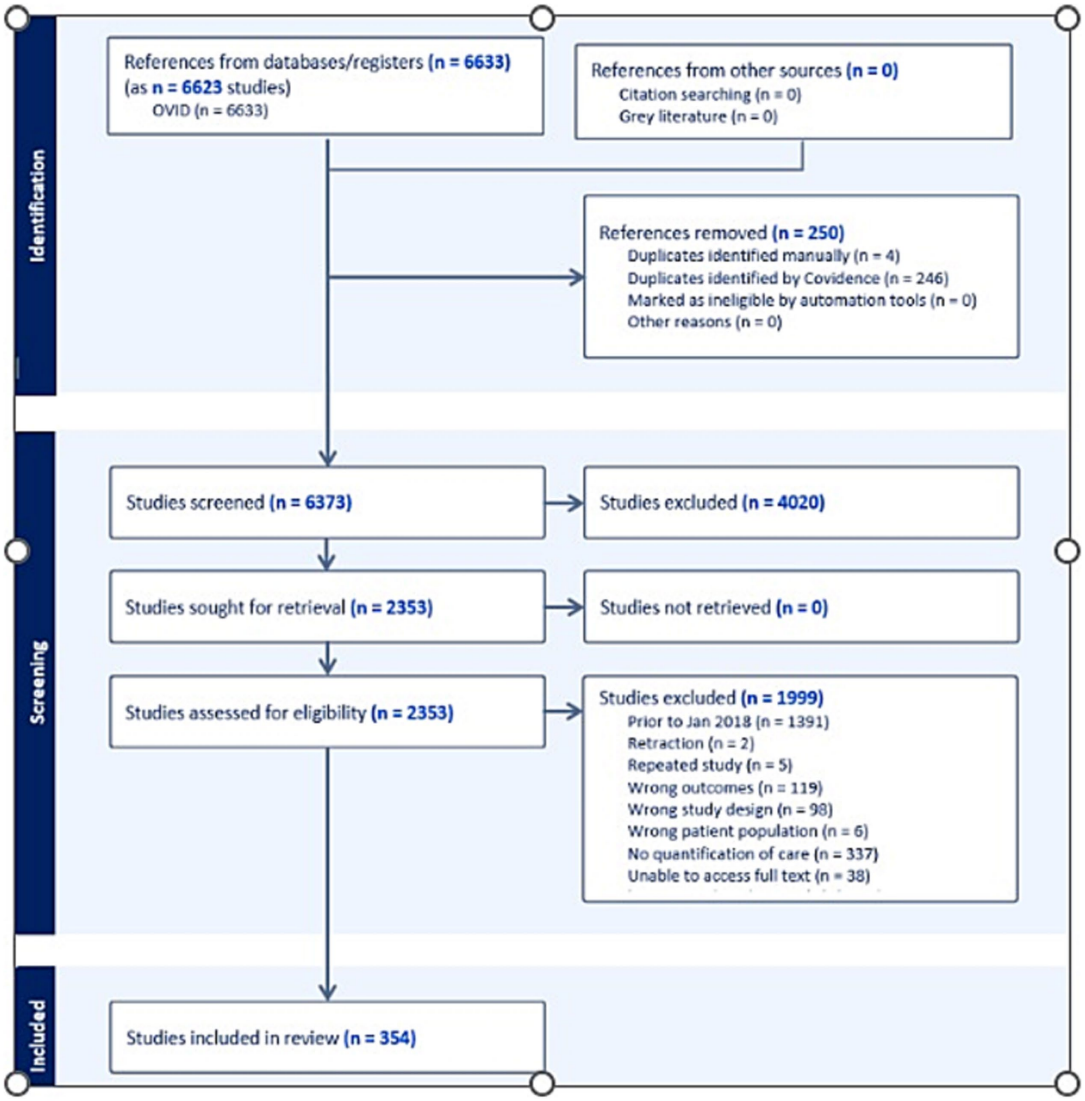
PRISMA diagram.

The initial databases searches identified 6,633 records. All records were entered into Endnote reference manager and Covidence where duplicates were removed resulting in 6373 records. Title and abstract screening resulted in the exclusion of 4,020 records, leaving 2,353 articles for full text screening, A total of 608 records were excluded due to the following reasons; retracted article (n-2), repeated study (*n* = 5), excluded outcomes (*n* = 119), excluded study design (*n* = 98), excluded patient population (*n* = 6), no quantification of care (*n* = 337), unable to access full text (*n* = 38) and studies not published in English (*n* = 3). In addition, 1,391 articles published prior to January 2018 were excluded. A total of 354 records were included in the final review, as shown in the PRISMA diagram in Figure 1.

### Scope and map the current state of the global care economy

**Figure 2:**
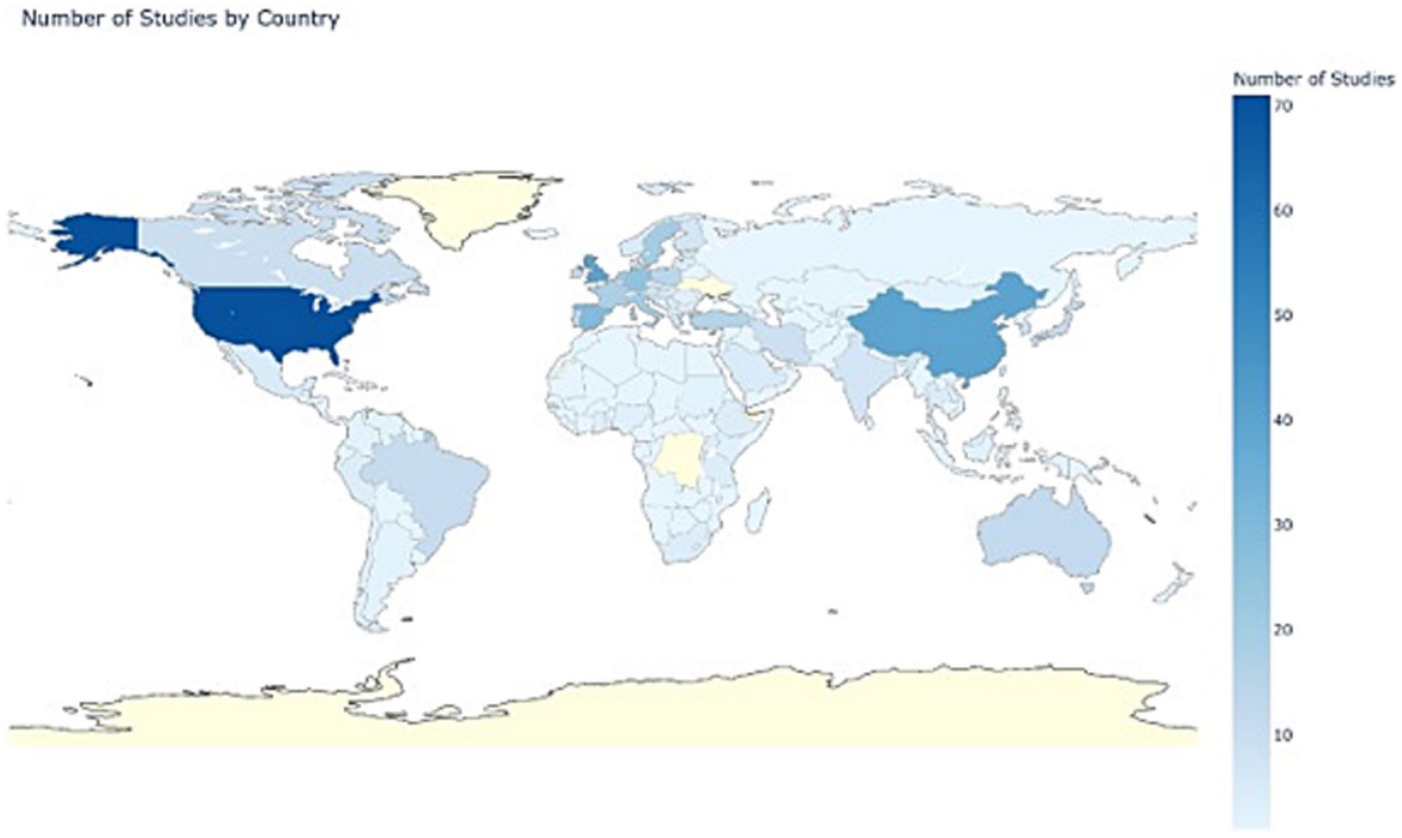
Number of studies by country.

There were 194 countries represented in the 354 studies. The highest representation was the United States (20%), followed by China and the United Kingdom (11% each). This distribution underscores a significant concentration of research in Europe and Asia, while other regions were underrepresented. There were 8% of studies that included multiple countries. The visual distribution of the number of studies by country is shown in Figure 2.

### Types of study design

Of the 354 studies, 60.0% were cross-sectional studies. Economic evaluation including cost analysis was attributed to 10.0% of the studies. Cohort and qualitative studies comprised 9.6 and 5.9% of studies, respectively. Thirteen studies were randomized controlled trials and 11 were of mixed methods design. The remaining study designs were case control (1.4%), discrete choice experiment, questionnaire and prevalence (1.1%), non-randomized and observational (0.8%), and only one multistage sampling study design.

### Care recipients’ characteristics

Table 2 shows the distribution of the care recipients and caring profile. Care recipient age groups included children (under 18), adults (18–64) and older people (65 and older). A quarter of the studies included adults. Older people aged 65 years and above were included in 25% of studies, while adults aged 18 to 64 years were included in 6% of studies. These age groups combined were included in an additional 27% of studies. The age of care recipients was not stated in approximately 27% of studies.

**Table 2:** Characteristics of caregivers and caring recipients.

Care recipient characteristics (*N* = 354)	n (%)
Adults (18 years to 64 years); Older persons (65 years and above)	97 (27.4)
Older persons (65 years and above)	89 (25.1)
Children (under the age of 18 years)	29 (8.2)
Adults (18 years to 64 years)	22 (6.2)
All age groups	15 (4.2)
Children and adults	7 (2.0)
Children and older persons	1 (0.3)
Not reported	94 (26.5)
Caregiver characteristics (*N* = 354)	
18 years to 64 years; 65 years and over	181 (51.1)
18 years to 64 years	88 (24.9)
Not reported	53 (15.0)
Under 18 years; 18 years to 64 years; 65 years and over	16 (4.5)
65 years and over	11 (3.1)
Under 18 years; 18 years to 64 years	4 (1.1)
Under 18 years	1 (0.3)

### Informal caregivers’ characteristics

The most frequently reported relationship to the care recipient included spouse or partner (60.2%) and adult children of the recipient (50.1%), followed by wider family network (35.0%), parents (26.6%), siblings (21.2%), friends or neighbors (16.1%), grandparents (5.1%), and children under 18 years of age (2.8%). Single parents (0.8%) and guardian (0.6%) accounted for a small number of studies. Studies that included only one type of informal relationship accounted for 11.6%. These included adult children, children under 18, parent, spouse or partner, other and wider family network. The remaining studies included a variety of relationships (68.6%), or the relationship was not reported (20%). Over 30% of studies did not specify relationships and often reported in the data as ‘other’. Where one caregiver relationship was reported, this was mostly as parents (3.7%), adult child (1.9%), spouse or partner (1.7%) and wider family network (0.6%). Grandparents were reported in one study as the sole relationship and one study reported children as the sole relationship.

### Formal care workforce characteristics

Formal care workforce, including care workers and health professionals, were underrepresented in the selected studies. Care workers or health professionals were included in 6.7% of studies combined with other relationships. While care workers or health professionals alone were represented in eight of the studies.

Multiple care relationships were reported in 6.7% of studies reported multiple care relationships including care workers or healthcare professionals, with nine studies including this group alone. Where studies included identification of care workers and professionals, these included early childhood education workers (0.3%), migrant care workers (1.7%), home care workers or health professionals generally (2.0%).

### General caregiving characteristics

The distribution of caregiver age is shown in Table 2. The age of caregivers was predominantly adults 18 years of age and older (51%), adults between 18 and 64 represented in 25% of studies. Most of the studies included both male and female caregivers (95%). There were no studies that included only male caregivers, while 5% studies included female only. The type of care provided by caregivers was predominantly both direct and indirect care (41.8%). This indicated that care was provided with the care recipient (including online or by phone) or conducting care related tasks on behalf of the care recipient. Direct care only (face-to-face) was specified in 18.1% of studies and indirect care only in two studies. The type of care delivery was not mentioned in 39.2% of the studies. Informal care was discussed extensively in 78% of studies, while 17.8% covered both formal and informal care.

The nature of caregiving was found to be long term in many studies (83.9%). Care was considered long term when the study included caring for those with a disability or chronic health condition including mental health conditions. Short-term care was included in 4.6% of studies and included caring for those with injuries or illness (short-term conditions). Both long and short-term care were included in 3.7% of the studies.

### Other types of care providers

Migrants as care workers were underrepresented in studies (3.1%). The capacity of migrant workers varied from formal foreign domestic workers 1.7%, to informal roles 1.4%. The informal roles of migrants were related to caring for their own family in addition to the formal caregiving role. Migrant live-in formal caregivers may suffer mental health impacts by being separated from their own families and being socially isolated. However, working as a caregiver was also found to contribute to good physical health [ID 44]. Migrants w more likely to be caregivers than non-migrants in some countries which care leads to a negative impact on their health, placing a further burden on the care and health system, these caregivers then have potential to be the care recipients in the future [ID 101, 158].

### Care settings

The care setting was primarily home-based (91.2%). Other care locations included hospitals (2.8%), residential care (1.7%), primary care or community-based (0.8%), more than one care setting was represented in 2.6% of studies. Where there were multiple settings identified in a study, home-based was included in all and was the focus of the study. Therefore, the total percentage of studies that included home-based setting was 93.5%.

### How care was funded

Care was mostly funded by caregivers themselves, this contributed to 64.1% of studies. Self-funding, in addition to public or private funding accounted for 15.3% of studies. Other funding methods such as public funding alone was provided in 3.7% of studies, private and not-for-profit or for-profit provided in 0.8% of studies. Clarity of funding other than self-funding was lacking in 15.2% of the included studies.

### Methods of quantification of care

Studies used a variety of ways to quantify care. Quality of life quantification was mostly demonstrated by using available validated tools, or specific tools created for the study in 68.6% of the studies. The most common tool used was the Zarit Caregiver Burden Interview (ZBI), which included items that address the financial burden of caregiving along with the psychological and physical costs of caring (31). The ZBI was also used to quantify the challenges caregivers face to their quality of life, commonly along with the Health-Related Quality Of Life (HRQOL) scale and the EuroQol (EQ-5D). Studies used a variety of ways to quantify care. Quality of life quantification was mostly demonstrated using available validated tools, or specific tools created for the study in 69% of the studies.

Quantification of time included time spent providing informal care or providing formal care 63.8%. How time was recorded, and the amount of time varied between studies, with some requiring a diary and others an estimation of time provided at data collection points in the study. Financial quantification included cost of health care, out-of-pocket costs related to health care, and costs related to provision of care in the care setting 34.2%. Quantifying employment included absenteeism and/or presenteeism, reduction in work hours and opportunity costs 25.1% for both formal and informal caregivers. How time was recorded, and the amount of time care was provided varied between studies, with some using a diary and others an estimation of time provided at data collection points in the study.

### Barriers and facilitators to care provision

Barriers to care provision were included in 94.9% of studies while facilitators were found in 81.4%. Two main themes that emerged were the psychological and quality of life impacts on caregivers and the cost of caregiving. Other emergent themes included impacts on the formal care workforce, gender and equality. The final theme related to the impacts of the COVID-19 pandemic on caregiving. The data extraction for the barriers and enablers is provided in Appendix 3, the themes and subthemes are shown in Table 3.

**Table 3:** Care economy themes and subthemes.

Theme	Barriers to care provision		Facilitators to care provision	
	Subthemes	Word count	Subthemes	Word count
Psychological and quality of life impacts on caregivers	health conditions/disease/ functioning/cognition/symptoms/ behaviors	394	psycho-educational/resources/ training/education/programs/information/skills	230
	burden	332	improving quality of life/wellbeing	152
	caring activity/tasks/intensity	94	social support/ leisure/community/religion	99
	fatigue/strain/stress/distress	70	resilience/mediating/fulfillment/religion	27
	depression	42	preparedness/ awareness/promote	26
	loneliness/isolation	18	strengthening/ advocate	9
	anxiety	16	psychosocial support/ counseling	9
	rural	14		
	low satisfaction/stigma	13		
	informal care hours/years/ time/labor	165		
Economy and the cost of caregiving	money/spend/ out-of-pocket/ expenditure	86	financial/funded/compensate/pension/insurance/Medicaid	176
	low socioeconomic	10	formal/paid/employment/income	144
	hardship/impoverished	7	physicians/ professionals/nursing	98
	labor/workforce/working	115	government/framework/policy/structures	49
	leave/h/productivity/absenteeism	86	availability of childcare	12
	career impacts	7	flexibility	7
	ethnic	3		
Gender and equality	females/women	85		
	gender	30		
	migrants and immigrants	11	empowering	2
	males/men	16		
	inequality	8		
	sandwich generation	6		
	discrimination	2		

### Psychological and quality of life impact on caregiving

The emotional and psychological demands on caregiving were identified as significant factors in the ability to continue to care. Spouses or partners who cared for people with dementia reported a higher degree of demand than other caregiver relationships [ID 176, 241, 243, 244]. The presence of abuse within family relationships contributed to the burden of care [ID 29, 227, 247, 305]. Where there was gratitude or appreciation expressed by the care recipient, the levels of demand lowered [ID 229, 298, 340]. Higher demands were found in familial relationships when caregivers lived with the care recipient, along with loss of leisure time and vacation time, or when behavior issues existed [ID 87, 170]. Parents and grandparents of unwell children experienced high levels of demand [ID 155], and this also had a flow-on impact on siblings. Where support was outsourced, an improvement in the quality of life for caregivers was seen [ID 103]. Once caregivers began to make use of formal care options, they were more willing to accept support [ID 164, 310]. Likewise, caregivers’ education led to a better quality of life and enabled them to deal with stressful situations and to access support and resources [ID 100, 173, 246, 270].

The longer caregivers were in the caring role, the more likely they were to experience caregivers’ burden and poor quality of life [ID 17, 111, 106, 150, 167, 178, 198]. Similarly, the amount of time spent (hours, days, years) on caregiving or completing unwanted tasks contributed to higher burden and poorer quality of life [ID 33, 46, 49, 73, 86, 118, 144, 204, 218, 251, 258, 262,326]. The higher the intensity of caregiving, i.e., higher needs (including time) of the care recipient, the higher the burden and its impact on quality of life and their unmet needs [ID 2, 8, 10, 42, 56, 60, 63, 75, 142, 150, 161, 187, 220, 217, 225, 228, 259, 261, 262, 279, 325, 296, 348]. Long-term care and high intensity care such as palliative, dementia, disabilities requiring full-time care, were shown to have negative physical and psychological effects on caregivers [ID 65, 108, 110, 115, 127, 173, 181, 332, 291, 338, 345].

Where care for people with serious illnesses occurred at home, support for family caregivers became more critical [ID 24, 74, 216, 237, 264, 315]. Loss of social support impacted on isolation and loneliness [ID 14, 43, 54, 112, 135, 152, 156]. Improving social networks for family and friends increased quality of life and reduced care burden [ID 282, 338, 346]. for example, leisure and social support activities, reduced depression level [ID 171,283, 291, 297, 328] or offered the option and time to pursue activities of interest [ID 145]. Providing caregivers with resources such as finance and food, along with psychological and intangible support and education, impacted positively on mental health, self-efficacy and well-being [ID 4, 21, 26, 55, 66, 77, 81, 85, 90, 102, 108, 139, 174, 185, 210, 224, 250, 253, 287, 318, 331, 335]. Some caregivers felt morally obligated to provide care but when the caring duties were shared, there were reduced feelings of loneliness [ID 137, 168, 252]. There are times when the caregiving role is not a choice, but rather a responsibility. When individuals do not have the choice to take on the caregiving role, it can affect the autonomy and decision-making power of both the caregivers and those receiving care [ID 83, 146, 203]. Mental health conditions were found to be associated with stigmatization [ID 54, 100]. While religion or faith-based support enabled coping mechanisms of caregivers [ID 132, 248, 252, 310]. Increased access to regular rest times, respite, social support and encouragement to request support were beneficial [ID 215, 262, 299, 248, 302, 314, 342].

Including caregivers and families in care planning was shown to be important not only for the health and well-being of the care recipient, but also to reduce caregiver burden in long term care [ID 13, 16, 170, 255, 274]. Education and communication provided to caregivers, eased care burden [ID 37, 48, 99, 123, 143, 184, 202, 215, 265, 294, 321, 350]. Studies that included education (such as resilience and safe manual handling) and support for caregivers had positive impacts on quality of life and longevity of caregiving [ID 116, 133, 191, 212]. Support for families through self-care, resilience or mediation programs could contribute to positive mental health [ID 7, 71, 233, 320].

Health care professionals’ engagement with caregivers and care recipients alleviated burden and improved patient outcomes [ID 153, 175, 191, 205, 232]. Training for the healthcare team was recommended to better identify needs and assess risks, and support caregivers [ID 11, 114, 117, 165, 169, 239, 250, 256, 344]. Where support was provided by health professionals this was associated with less caregivers’ stress [ID 57]. Training and support for formal caregivers in the case of high intensity support had potential to overcome care barriers [ID 21, 22, 26, 32, 88, 104, 174, 234, 250, 318, 335]. Follow-up phone calls after discharge or after primary care appointments were found to reduce burden [ID 15, 110, 122, 38, 169].

Assistive technology was shown to provide the care recipient with higher independence [ID 53, 66, 63, 69, 120, 230, 280], although the set-up costs were deemed expensive [ID 84]. Innovative humancentric technology that was designed to assist caregivers’ knowledge of health conditions and behaviors supported caregivers’ management of stress [ID 93, 105,134, 267, 324]. Technology could support a person-centered approach and minimize the use of pharmaceutical treatments [ID 105, 134].

### Time, cost and the impact of caregiving

The cost of care further worsened the loss of income for caregivers. It was common for caregivers to bear out-of-pocket costs which contributed to higher financial burden and employment changes [ID 47, 68, 70, 121, 124, 159, 177, 213, 26]. The out-of-pocket costs included taxis and other transport, parking and food at hospitals, accommodation, personal items such as continence aids [ID 95, 96, 201, 289, 304, 312, 316]. Services such as ride sharing had the capacity to reduce transport costs and caregiver burden and contribute to social support for caregivers and recipients [ID 203]. Unsurprisingly, higher income was found to be related to higher out-of-pocket expenses [ID 208, 303]. Time for caregiving was also found to increase financial strain [ID 8]. In addition to time, frailty also contributed to higher cost for care as more tasks and support for the care recipient were needed [ID 6, 8]. Where caregivers had dependents in addition to the care recipient, there was less income available from formal work placing them at a greater risk of poverty [ID 31, 323, 275]. Single parents experienced higher financial burden due to reduced family income [ID 51, 268]. Grandparents suffered a worsening financial situation by giving up work to care for grandchildren [ID 194]. Caring duties hampered the capacity to be steadily employed and for professional growth [ID 137, 149]. Understanding the perspectives of parents when making policy regarding childcare was important to ensure the right improvements were made [ID 136]. Childcare support provided a protective element to the well-being of a family caregiver where children were care recipients [ID 163, 272, 285].

Workforce impacts for informal caregivers were often identified as loss of productivity and absenteeism [ID 18, 45, 61, 79, 80, 92, 98, 126, 148, 157, 172, 182,192, 226, 242, 271, 273, 281, 329, 330, 337]. Factors that influenced absenteeism were cognitive impairment of the care recipient [ID 89] and short-term illness and end-of-life care [ID 109, 295]. Home care workers were more likely to experience depression if they suffered an economic burden, had a poor relationship with the client or were subject to discrimination [ID 349].

Only half of higher-income countries were more likely to provide formal leave for caregivers [ID 126] and with a shortage of doctors in some countries it could be difficult to access the required medical certificates to enable formal care days off from work [ID 1]. Full-time caregiving resulted in caregivers needing to leave formal employment [ID 137, 209, 323]. Caregivers were often in manual labor roles as they had more flexible hours, however this added to the physical burden of care [ID 50, 137, 288]. Higher levels of care duties, for, e.g., longer duration of time, resulted in less time available for employment and therefore led to the risk of financial impoverishment [ID 131, 183, 334].

For caregivers, the loss of income or employment was found to be linked to their worsened quality of life more than the severity of disease itself [ID 141]. Households in rural areas with male patients were more likely to have less income than those with female patients, likely due to the males being employed while the females performed unpaid housekeeping duties [ID 341].

The overall economic value of informal care exceeded the cost of formal care in many studies [ID 76, 129, 200, 222]. Informal caregivers were more financially vulnerable than formal workers [ID 130, 219, 276, 311]. This vulnerability included insecurity of food, home ownership, and their own health needs. Those who were in a better financial position were noted to be more likely to access formal support and care [ID 3, 12, 23, 41].

Informal caregiving came at a cost and high financial burden particularly for rural caregivers, however, rural caregivers had additional coping mechanisms, such as identifying informal supports and thus contributing to lower caregiver burden [ID 28, 59, 147, 162]. Caregivers who experienced economic strain were more likely to engage in maladaptive behaviors. Screening for financial difficulties could identify and alleviate potential risk factors [ID 82]. Rural caregivers experienced significant opportunity costs and unmet need in their caring role and less access to services [ID 190, 151, 327, 336]. This was impacted by the caregivers missing out on career opportunities [ID 72, 78, 128, 223, 266, 286, 301, 313, 322, 347]. Added to this was the emotional and financial burden of the amount of support provided not being enough to cover the real costs of caring [ID 17, 45, 88, 113, 119, 160, 195, 196, 235, 236, 240, 245, 266, 269, 270, 290, 300, 307, 317, 351].

Some studies explored financial support for caregivers and how this could relieve burden. Financial subsidies and opportunities, when accepted or accessed, could support caregivers in relieving burden [ID 34, 52, 111, 231, 299, 303, 308]. Improved social support systems, early support interventions and providing long-term care insurance were recommended [ID 19, 97, 332]. The support of financial subsidies and supportive workplaces could contribute to caregivers being more able to remain at work on reduced hours [ID 9, 111, 150]. While some studies suggested that financial support, such as Medicaid in the United States were required, other studies suggested that support through an allocation of care or counseling [ID 25, 26, 39, 94, 319, 254] could provide some relief. Australia’s home care packages (HCP), where a government allocated a level of funding, ensured that support and clinical care were provided at home [ID 322].

### Gender and equality

Barriers and facilitators for women as caregivers was a focus in 24% of studies. Women were more likely to be impacted by loss of income, increased debt, and caregiver burden due to the lack of centralized coordination of both short-and long-term care relief options [ID 4, 181, 189, 193, 199, 211, 212, 223, 238, 278]. Women were also more likely to have multiple caring roles within the family unit, this contributed to their higher burden compared to men [ID 40, 91, 154, 166, 188, 284, 306, 323]. Older working, female caregivers had the additional burden of their own declining health [ID 119, 263] and higher perception of unmet need [ID 293]. Working women were found to also have more caregiving duties, suffer higher personal and family stress in additional to their formal work responsibilities [ID 36]. Women and workers over 50 years of age were more likely to have informal care roles on top of formal work [ID 126]. Children as caregivers could be impacted by reduced education opportunities [ID 43, 140].

Some countries like Germany, Italy, and Ireland found that men were less inclined to reduce employment hours to provide care, while women were more likely to take on the caring role [ID 78]. Generous long-term care (LTC) policies that include welfare for caregivers could provide the opportunity to include external services to support the caring role, enabling the caregiver to remain in formal work. This may attract more males to the caring role [ID 292].

Men and women responded differently to the caring role; further studies should consider the gender differences of social support perceptions [ID 125, 180, 206, 249, 257, 272, 352]. Men were found to be more likely to use external services to support a care recipient with dementia than women [ID 309]. Younger age, women, ethnic minority women [ID 30, 186] or lower socioeconomic groups [ID 58, 214, 221], had poorer mental health and higher social isolation, which had a negative impact on their quality of life [ID 277]. Racism could impact the types of roles formal caregivers would be successful in getting [ID 20].

### Impact of the COVID-19 pandemic on caregiving

The review included studies pre-and post-COVID-19. While COIVD-19 itself was not a specific focus of the study there were some notable findings. The COVID-19 pandemic resulted in cessation of many supports impacting the availability of respite and downtime for caregivers and childcare services for employees [ID 62, 107]. Services such as domiciliary and early childhood and education centers should develop contingency plans for emergencies or pandemics, e.g., the COVID-19 pandemic to ensure continuity of service [ID 27, 35, 67, 138, 207]. The pandemic also increased the reliance on informal care options, which resulted in physical and mental detriments to caregivers, impacted on finances and quality of life, and highlighted the need for continuing care support [ID 5, 92, 107, 197, 354].

## Discussion

This is the first scoping review to examine the current state of play of the care economy globally. The review identified the scholarly treatment of the concept of ‘care economy’ across the life course. The total number of published papers that met our criteria was high (354 in the past 5 years); this is unsurprising given the breadth of the care sectors that the care economy represents. Yet our scoping review demonstrated paucity of evidence particularly from countries such as New Zealand, Africa, Australia, Canada and Latin America. This is despite the fact that the care economy is the largest and fastest growing sector across the globe (23). Indeed, care continues to be contested territory as to who is responsible for delivering and paying for care. In some countries, care falls within the formal or informal sector or the welfare system (32).

### Contemporary evidence on the care economy

This review found that women continued to be overrepresented in caregiving (33). The burden of care is disproportionately higher for older people and predominantly women (3, 34). Women played a critical role in caring duties for children and grandchildren (35), while men were more likely to have chronic diseases (36). The high contribution of women to caregiving duties had a flow-on effect to unemployment and their ability to earn an income (37).

During the COVID-19 pandemic, with entire families being housebound due to lockdowns, the care work performed by women including cooking, cleaning, laundry and supervisory activities performed was exposed (15). Women were negatively impacted by COVID-19 in relation to finances, mental health and time for caring in a study conducted during the pandemic by (38). The reliance on informal caregivers increased significantly during the pandemic and has since subsided according to a European study (39). While the reliance on informal caregivers may have returned to pre-COVID-19 levels there is a lasting impact on the cost-of-living (40). This study reported that there was an increase in wage inequity for low-income earners, particularly for women. The effects of COVID-19 are still being felt by low-and middle-income countries where the economy and employment have not yet recovered to previous levels (40).

We found that the contemporary evidence focused on the burden of care and quality of life for informal caregivers rather than the economic aspects of care. Defining the boundaries of the caregiving role will be important as future care responsibilities are shared by both the formal care workforce and informal caregivers. Traditionally, informal caregivers, often family members, provided personal care such as dressing, bathing and feeding (41). However, there has been a growing trend where these tasks are increasingly performed by home care workers (42, 43). This change is driven by various factors, including the rising demand for care for older people, the complexity of care needs and the availability of home care services (43). On the other hand, there is higher expectation for informal care to coordinate and deliver specialized care such as palliative care at home (44). In highlighting the level of burden or quality of life, education for both formal and informal caregivers was frequently suggested (45). This education could include emotional well-being, resilience, or skills-based training (such as hygiene). Our review found that most studies included informal caregivers as a focus to highlight the hidden costs of care. As the population ages the reliance on formal and informal care will increase (46). In order to support people to receive care at the right place and at the right time, policy makers need to develop sustainable care and social care models (47). These models include financial support such as welfare and insurance systems, and a greater recognition of the link and interaction between formal and informal care and to promote economic empowerment. In addition, policy needs to support informal caregivers who are often in paid employment, to facilitate flexible leave and working conditions (48).

Caregivers in some cultures experienced a sense of reward from providing care, due to cultural expectations, familial duty and a sense of responsibility for older adult’s care (49). Migrants and minority groups continued to be underrepresented in studies. Studies that did include migrants did not strongly represent them as a focus of the paper (50). In addition, racial and ethnic caregiver groups were deemed to be underrepresented in policy reviews in public health crises. More research is suggested in disadvantaged or vulnerable population groups. Care-friendly policies such as International Organization for Standardization (ISO) quality standards for workplaces should be considered that support migrants who are providing transnational care (51).

Cross-sectional study design was identified in the studies as a limiting factor in the analysis because of the single point data (52). Longitudinal studies can better elucidate the ongoing impact of informal caregiving on quality of life. Aspects such as capturing the complexities of caregiving and employment decisions, and caregiver support strategies to relieve burden and the impact of care intensity on the caregiver can be more effectively documented and monitored over time (53).

### Education for formal care workforce and informal caregivers

More information about the types of care tasks performed would elucidate where caregiving time is spent and what support or education is required (43). The non-healthcare cost associated with caregiving needs to be examined to fully understand the burden of disease on caregivers (54). Understanding the daily living requirements and unmet needs of families in the case of children with long-term conditions or chronic diseases is important to ensure the family is supported. Objective time measures, such as diaries, could be used to determine caregivers’ unmet needs which could then inform support or education (55). Improving the scope of practice and diversity of formal care workers, including psychological training, could increase the quality of care delivered. Culturally sensitive interventions to support caregivers are needed for their own physical and psychological health care needs (56). Education provides skills for caregivers in addition to improving respite options. Research on gender differences in caregiving is required in order to consider the ecology of family caregiving including racial/ethnicity impacts and caregiving time in the social care system.

Regulatory accountability in training and credentialing for formal carers is vital but often inconsistent (57) and formal carers in developing countries have even less access to education and training resources (58). There is also an increasing focus on the need for informal carer training, as they are often caring for people with chronic and debilitating conditions that require knowledge and skills in manual handling and hygiene, along with much resilience. The World Health Organization has developed an iSupport training program for carers of people with dementia, which can be culturally adapted to national or local settings, and carers can complete all modules and lessons sequentially or choose the most relevant ones and proceed at their own pace, with feedback provided upon completion (59). Such programs that can be customized and distributed online would lead to the possibility of greater reach and less variability of training and resources for both formal and informal carers across countries.

### The economics of care

Our review highlighted the lack of funding models and policy toward a sustainable care ecosystem. Governments need to explore avenues to support caregivers and families financially beyond the welfare system (60). There needs to be a greater understanding of the link and interaction between formal and informal care and to promote economic empowerment. Policies are required to support families and reduce gender inequality in informal care; supporting this would improve care in children. Further examination of the labor market and economic evaluation may cover discrimination of gender, caregiver role, diversity, caring role and impact on caregivers, impacts of caregivers and care recipient choice and control. The reliance on informal care will be impacted by the shrinking caregivers supply, as the population ages and low births rate exist. Governments need to develop sustainable care models to support the growing demand for home-based care (61). Policies should consider supporting family caregivers, particularly the ‘sandwich generation’ wherein caregivers provide care to both children and older adults (62). The impacts of caregivers should be included in any out-of-pocket costs and cost-of-illness analysis to demonstrate that the disease burden is more than the disease alone. Young caregivers need support to ensure they are not educationally disadvantaged. It is important to consider reducing overreliance on family and increasing the support for family caregivers.

Interventions aimed at maintaining or improving relationships between the care recipient and the caregiver while enhancing caregiving dynamics, have the potential to improve the perception of social support and lower burden (63). Additionally, labor market productivity, informal care costs, and cost of illness evaluations should be conducted when determining cost-effective interventions for diseases (64). The economic impacts of the burden of disease need to include consideration of the health-related quality of life for informal caregivers.

The societal impacts on both the caregiver and care recipient should be considered when exploring financial security options, particularly for long-term home-based care (65, 66). Indirect and direct costs that impact productivity losses and how this influences the socioeconomic status of caregivers and care recipients should also be considered (54). Characterizing and measuring the care economy brings significant economic and social benefits. It increases visibility for both formal and informal care work, often undervalued in traditional economic metrics, allowing policymakers to make more informed decisions. This recognition helps integrate the care economy into national accounts, making it a formal part of the economic plan. Moreover, measuring the care economy highlights gender disparities, as women disproportionately bear the burden of informal care work. By addressing these inequalities, governments can develop policies that promote gender equality and fair compensation for care work (23).

### Strengths and limitations

Our research question was intentionally broad as we would like to gain an insight into this emerging area. As we selected a large number of studies with a broad variety of topics, we were unable to review each study in more depth. A limitation is the variability of search terms across populations and countries. Despite the search terms we have used, there may still be publications missed. We have used multiple terms to capture different populations and settings such as paid, unpaid, formal and informal. We have not included the term aged care as it sometimes is often treated as a distinct policy and funding area, with specific regulatory frameworks, workforce needs, and service models that may not align with broader care economy discussions. This study was limited to health-related search engines, thus there may be a bias toward health in the selected studies. This was shown in the large number of disease or condition focused studies. Future research could cover a more diverse range of databases. We did not consider the dropout rate of participants in studies; this was found incidentally during our analysis. Some studies explained the dropout was due to caregivers’ perception of increased burden by participating in the study. Engaging caregivers in studies can be difficult, researchers need to find a way to support caregivers to ensure their valuable contribution to research is sustained during the study period.

## Conclusion

The care economy gained its prominence following the COVID-19 pandemic. The evidence mostly focused on the role of informal caregiving and the negative impacts of caregiving. Examining the value of care both in formal and informal sectors is critical to sustain care demands against depleting supply of care provision globally. Governments around the world are now investing and setting up policies to tackle the challenges of delivering care given the demographic shifts and rise of living costs. More innovative research is needed in the care workforce of the future where technology will play a significant role in care delivery.
