# Supplementary material for: What is the care economy? A scoping review on current evidence, challenges, facilitators and future opportunities
Source: Front Public Health. 2025 May 14;13:1540009. doi: 10.3389/fpubh.2025.1540009 (PMC12116328; doi:10.3389/fpubh.2025.1540009)
Supplement: Supplementary file 1 [file Data_Sheet_1.docx]

**Appendix 1 Database search strategy**

Database: JBI EBP Database <Current to October 11, 2023>, Embase Classic+Embase <1947 to 2023 October 18>, Ovid MEDLINE(R) ALL <1946 to October 18, 2023>, APA PsycInfo <1806 to October Week 2 2023> Search Strategy:

--------------------------------------------------------------------------------

1 (Cost$ or economic cost$ or economic burden or expenditure$ or economy).mp. [mp=tx, hw, sw, ti, ab, tn, ot, dm, mf, dv, kf, fx, dq, bt, nm, ox, px, rx, ui, sy, ux, mx, tc, id, tm] (2662898)

2 care economy.mp. [mp=tx, hw, sw, ti, ab, tn, ot, dm, mf, dv, kf, fx, dq, bt, nm, ox, px, rx, ui, sy, ux, mx, tc, id, tm] (301)

3 Caregivers/ or care economy.mp. (180732)

4 delivery of health care.mp. or "Delivery of Health Care"/ (328553)

5 social support.mp. or Social Support/ (336698)

6 1 and 3 and 4 (779)

7 1 and 3 and 5 (2874)

8 6 or 7 (3570)

9 2 or 8 (3830)

10 remove duplicates from 9 (2658)

11 limit 10 to english language [Limit not valid in JBI EBP Database; records were retained] (2481)

12 limit 11 to human [Limit not valid in JBI EBP Database; records were retained] (2398)

13 limit 12 to humans [Limit not valid in JBI EBP Database,APA PsycInfo; records were retained] (2398)

***************************

**Database:**
Ovid MEDLINE(R) ALL <1946 to October 20, 2023>

| **#** | **Query** | **Results from 24 Oct 2023** |
| --- | --- | --- |
| 1 | (Cost* or economic cost* or economic burden or expenditure* or econom* or care economy).mp. [mp=title, book title, abstract, original title, name of substance word, subject heading word, floating sub-heading word, keyword heading word, organism supplementary concept word, protocol supplementary concept word, rare disease supplementary concept word, unique identifier, synonyms, population supplementary concept word, anatomy supplementary concept word] | 1,499,932 |
| 2 | Caregivers/ | 50,743 |
| 3 | carer*.mp. | 19,292 |
| 4 | 2 or 3 | 62,982 |
| 5 | 1 and 4 | 10,865 |
| 6 | ((paid or unpaid or informal or non profit or "not for profit") adj3 (work* or employ* or labour or car*)).mp. [mp=title, book title, abstract, original title, name of substance word, subject heading word, floating sub-heading word, keyword heading word, organism supplementary concept word, protocol supplementary concept word, rare disease supplementary concept word, unique identifier, synonyms, population supplementary concept word, anatomy supplementary concept word] | 16,969 |
| 7 | 4 and 6 | 6,753 |
| 8 | 1 and 7 | 1,855 |
| 9 | ((care* or caring) adj2 (family or home or paid or unpaid of informal or non profit or "not for profit" or work*)).mp. [mp=title, book title, abstract, original title, name of substance word, subject heading word, floating sub-heading word, keyword heading word, organism supplementary concept word, protocol supplementary concept word, rare disease supplementary concept word, unique identifier, synonyms, population supplementary concept word, anatomy supplementary concept word] | 122,287 |
| 10 | 6 or 9 | 134,620 |
| 11 | 4 and 10 | 21,926 |
| 12 | 1 and 11 | 4,531 |
