# Supplementary material for: What is the care economy? A scoping review on current evidence, challenges, facilitators and future opportunities
Source: Front Public Health. 2025 May 14;13:1540009. doi: 10.3389/fpubh.2025.1540009 (PMC12116328; doi:10.3389/fpubh.2025.1540009)
Supplement: Supplementary file 2 [file Data_Sheet_2.docx]

**Appendix 2 Definitions of terms**

**Direct care** refers to care that is provided face-to-face or via telehealth with the care recipient present, for example hygiene, transport or accompanying medical appointments.

**Indirect care** refers to activities or tasks that are provided on behalf of the client, for example running errands, paying bills.

**Formal care** refers to the provision of care that is paid, for example home care workers, or health professionals.

**Informal care** refers to the provision of care that is unpaid, often provided by family members, relatives, friends, neighbors

**The care economy** represents diverse paid and unpaid work activities that provide direct and unpaid care for people needing support. This includes children, people with disabilities, and those who are elderly or sick. Provision of care includes self-care to support people to function capably, comfortably, and safely
